# Personality disorder functioning styles and empathy in trainee nurses: the mediating and moderating roles of death attitudes

**DOI:** 10.3389/fpsyt.2025.1532940

**Published:** 2025-03-27

**Authors:** Jianing Pan, Haozhen Wu, Yujie Wang, Bingren Zhang

**Affiliations:** ^1^ School of Clinical Medicine, Hangzhou Normal University, Hangzhou, China; ^2^ School of Nursing, Hangzhou Normal University, Hangzhou, China

**Keywords:** personality disorder functioning style, death attitude, empathy, mediating effect, moderating effect, trainee nurse

## Abstract

**Background:**

There is abundant evidence that an individual’s personality traits may influence their level of empathy. However, the role of death attitudes in the relationship between personality disorder functioning styles of nurses and their empathy remains to be elucidated.

**Methods:**

Personality disorder functioning styles, death attitudes, and empathy levels were assessed in 614 Chinese trainee nurses using the Parker Personality Measure (PERM), the Death Attitude Scale-Revised (DAP-R), and the Jefferson Scale of Empathy-Health Professionals (JSE-HP).

**Results:**

Among the trainee nurses, DAP-R Fear of Death, Death Avoidance, Escape Acceptance, Approach Acceptance, and Neutral Acceptance(-) partially mediated the negative correlations between all PERM styles except Obsessive-compulsive style and empathy. Furthermore, DAP-R Fear of Death, Escape Acceptance, and Approach Acceptance fully mediated that between Obsessive-compulsive style and empathy. Fear of Death and Death Avoidance attenuated empathy among those with higher Narcissistic and Dependent styles, respectively.

**Conclusion:**

Death attitudes served as a mediator and moderator in the relationships between personality disorder functioning styles and empathy among trainee nurses, suggesting the need for targeted death education among early-career nurses with personality dysfunctions.

## 1 Introduction

Empathy in clinical practice involves recognizing and experiencing a patient’s emotions (emotional empathy), understanding their thoughts and feelings (cognitive empathy), and effectively communicating this understanding to the patient (behavioral empathy) (1, 2). Whilst empathy (particularly its emotional aspect) is susceptible to innate factors, it can also be developed through intellectual processes such as transpersonal thinking, enabling nurses to understand patients while maintaining objectivity (3–5). Clinical empathy boosts nurse engagement (6), strengthens the nurse-patient relationship (7), and is crucial for holistic care (1). Moreover, it enhances healthcare workers’ personal fulfillment (8) and protects against burnout (9). Empathy tends to be high in early adulthood (10), but gradually declines with age, particularly in emotionally demanding jobs (11). Studies show that nurses worldwide are increasingly experiencing compassion fatigue—characterized by depletion or absence of empathy and is often accompanied by burnout (1, 12, 13), especially in high-stress settings like ICUs (14). Similar trends of decreasing empathy have been observed in nursing students in the US and Italy among those with increased clinical exposure (15, 16), and even among those with limited clinical experience (17). These findings suggest that, in addition to external factors like work environment and workload, intrinsic factors also play a significant role in shaping empathy in early-career nurses.

Literature has showed that an individual’s capacity for empathy is closely linked to their personality profile (18). Using the Big Five personality model, one of the most influential personality theories, studies found that personality traits account for 15.1%-37.5% of the variation in empathy (19, 20). To be more specific, positive associations were found between conscientiousness (19–22), agreeableness (19–21, 23), openness (21, 23), and extraversion (21, 22) and empathy, while a negative association was found between neuroticism and empathy (19, 22) in undergraduates, medical students, and clinical nurses at different career stages. However, less attention has been given to personality disorder functioning styles (disordered personality traits), which deviate from the norm and may evolve into established personality disorders (24). According to the DSM-5, personality disorder functioning styles are classified into three clusters: Cluster A includes eccentric traits such as paranoid, schizoid, and schizotypal personalities; Cluster B is characterized by dramatic, impulsive, and moody traits, seen in borderline, antisocial, narcissistic, or histrionic personalities; and Cluster C involves anxious and fearful traits, represented by avoidant, dependent, or obsessive-compulsive personalities (25, 26). Prior research has indicated that some nursing students and trainees are particularly susceptible to stress and negative emotions, particularly in the context of the COVID-19 pandemic (27, 28), increasing the risk of compassion fatigue (29). Meanwhile, in many cases, high accessibility to stress and negative emotions was associated with personality dysfunctions (25). Therefore it is highly possible that nurses with personality disorder functioning styles have lower levels of empathy than healthy ones. Interestingly, some evidence has suggested conflicting conclusions. For example, narcissistic style, marked by apathy, distrust, and interpersonal dissociation, has been demonstrated to be negatively correlated with empathic concern (30). However, a study in Berlin (31) found that while individuals with narcissistic personality disorder had deficits in emotional empathy, their cognitive empathy remained intact. This suggests that personality disorder functioning styles may not universally reduce empathy, with other cognitive factors possibly mediating this relationship.

An individual’s attitudes toward death are shaped by their comprehensive conceptualization of death, encompassing their own mortality and that of others (32). People with positive attitudes toward death can deal effectively with the negative emotions associated with death, whereas people with negative attitudes often show fear and avoidance of death, or see death as a means of escaping reality (33, 34). Evidence showed that COVID-19 exacerbated fear of death, while those exposed to high levels of epidemic-related media content showed higher levels of empathy or sympathy (34). Death exposure and fear of death were also found positively correlated with attitudes toward the care of the dying among certain percentage of residents (35) and nursing students (36). However, for some healthcare workers and students, such exposure leads to a sharp decline in empathy (35, 37, 38), which might be correlated with their personality dysfunctions to some degree. Additionally, some patients with schizophrenia, who are at higher risk for suicidal behavior, exhibit diminished fear of death and heightened empathy (39). Such findings indicated that individuals with mental illness may experience a unique relationship between death attitudes and empathy compared to those in good health. Additionally, death attitudes differ across personality disorders. For instance, narcissistic personality disorder was found associated with greater fear of death and less suicidal ideation, whereas antisocial and borderline personality disorders were linked to higher rates of suicidal thoughts (40, 41). Therefore, it can be proposed that personality disorder functioning styles may influence empathy in trainee nurses through mediation of their death attitudes.

In addition, death attitudes have the potential to moderate the negative prediction of empathy by personality disorder functioning styles. For example, participation in end-of-life care courses has been shown to increase empathy among undergraduates (42). There is also evidence that individuals with borderline personality disorder, who often struggle with chronic self-harm ideation and high suicide rates (41), typically exhibit low empathy levels (43). While following the psychologically oriented treatment, these patients exhibited a decline in suicidal self-harm ideation (44) and an enhancement in empathy (45).

In general, previous studies indicated that empathy was associated with personality traits (18–22, 30, 31) and attitudes toward death (34–38). However, the precise role of death attitudes in the relationship between personality disorder functioning styles and empathy in trainee nurses remains unclear. Clarification of this will enrich theories about the mechanisms of personality influence on empathy, and facilitate the development of a targeted joint empathy and death education training program for early-career nurses in need. We hypothesized that: 1) personality disorder functioning styles are negatively associated with empathy in trainee nurses; 2) these styles reduce empathy through the mediation of negative death attitudes; 3) death attitudes moderate the relationships between personality disorder styles and empathy.

## 2 Methods

### 2.1 Participants

A total of 626 trainee nurses from a general hospital in Hangzhou, China, were initially invited to participate in the anonymous online survey. A total of 614 valid responses were ultimately obtained (556 female and 58 male; mean age 21.85 ± 1.12, range 19 ~ 26 years). The remaining 12 responses were excluded on the grounds that they exhibited a standard score in excess of 65 on the Lie factor of the Parker Personality Measure, thereby indicating a low degree of accuracy in self-report. All the nurses were self-reported to have no history of psychiatric or neurological abnormalities, and they were not currently suffering from any acute or chronic illnesses. All participants were of Han Chinese ethnicity, had been engaged in hospital practice for a period of less than six months, and were assigned to rotate between different departments. **Table 1** presents their demographic characteristics and corresponding comparisons of empathy levels. The study was approved by a local ethical committee (No. 2024029) and all participants gave their informed consent to participate.

**Table 1:** Demographic characteristics and their relationships with empathy levels among trainee nurses (N = 614).

Variables	n (%)	JSE-HP total score		t/r/F	P
		Mean	S.D.		
Gender				t = -0.15	0.878
Male	58 (9.4)	116.38	12.78		
Female	556 (90.6)	116.66	13.16		
Age (years)				r = -0.14	0.001
19	4 (0.7)	114.00	12.44		
20	32 (5.2)	120.12	11.10		
21	220 (35.8)	119.34	11.87		
22	220 (35.8)	115.03	14.03		
23	103 (16.8)	113.65	12.40		
24	16 (2.6)	115.69	15.04		
25	10 (1.6)	117.90	17.14		
26	9 (1.5)	112.67	15.18		
Education level				F = 0.81	0.446
College student and below	54 (8.8)	118.80	14.14		
Undergraduate student	542 (88.3)	116.43	12.91		
Master candidate	18 (2.9)	116.17	16.22		

JSE-HP, the Jefferson Scale of Empathy-Health Professionals.

### 2.2 Questionnaires

#### 2.2.1 The Parker Personality Measure (PERM)

The PERM (26) involves efficient and first-level clinical description of personality disorder types Paranoid, Schizoid, Schizotypal, Antisocial, Borderline, Histrionic, Narcissistic, Avoidant, Dependent, Obsessive-compulsive, and Passive-aggressive when the standardized scores for the dimensions are greater than 65. It contains 92 items, each item is scored on a 5-point Likert scale (1 = very unlike me, to 5 = very like me). Studies have shown that the Chinese version of PERM can help measure personality disorder functioning styles in clinical practice with good reliability and criterion validity (46, 47). Its Cronbach’s alpha in this study was 0.82.

#### 2.2.2 The Death Attitude Profile-Revised (DAP-R)

The DAP-R is a questionnaire designed to measure attitudes toward death (32). It consists of 32 items divided into five subscales: Fear of Death, Death Avoidance, Escape Acceptance, Approach Acceptance, and Neutral Acceptance. Each item is scored on a 5-point Likert scale (1 = strongly disagree, to 5 = strongly agree). The Chinese version of the DAP-R has been shown to be reliable and valid (48), and its Cronbach’s alpha in this study was 0.88.

#### 2.2.3 The Jefferson Scale of Empathy-Health Professionals (JSE-HP)

The JSE-HP is a widely used instrument for measuring empathy in the context of health professional education and patient care (49). It has three dimensions, namely Perspective taking (ability to understand the patient’s perspective, reflecting cognitive empathy), Compassionate care (expression of caring, concern, and empathy), and Walking in patient’s shoes (feeling what the patient is feeling, reflecting emotional empathy). Items are scored on a 7-point Likert scale (1 = strongly disagree, to 7 = strongly agree), with half of the items reversely scored. The similarity of the JSE-HP patterns in samples from different countries suggested that the scale was consistently reliable and valid regardless of cultural differences (50), and its Cronbach’s alpha in this study was 0.75. However, we only used its total score for analyses, because from a clinical perspective, improvement in composite empathy was actually more valuable than that in its individual dimensions.

### 2.3 Data analysis

Statistical analysis was performed using SPSS IBM Statistics v.26.0. Descriptive statistics were used to describe demographic variables such as age, gender, and education level of participants. Comparisons of the composite empathy scores between participants of different gender and education levels were made using Student’s t-test and one-way ANOVA, respectively, and the correlation between empathy and age was tested using Pearson correlation analysis. The significance of the P value was set at < 0.05. Once the influence of demographic variables on total JSE-HP scores was identified, these were used as covariates in further analyses.

**Figure 1:**
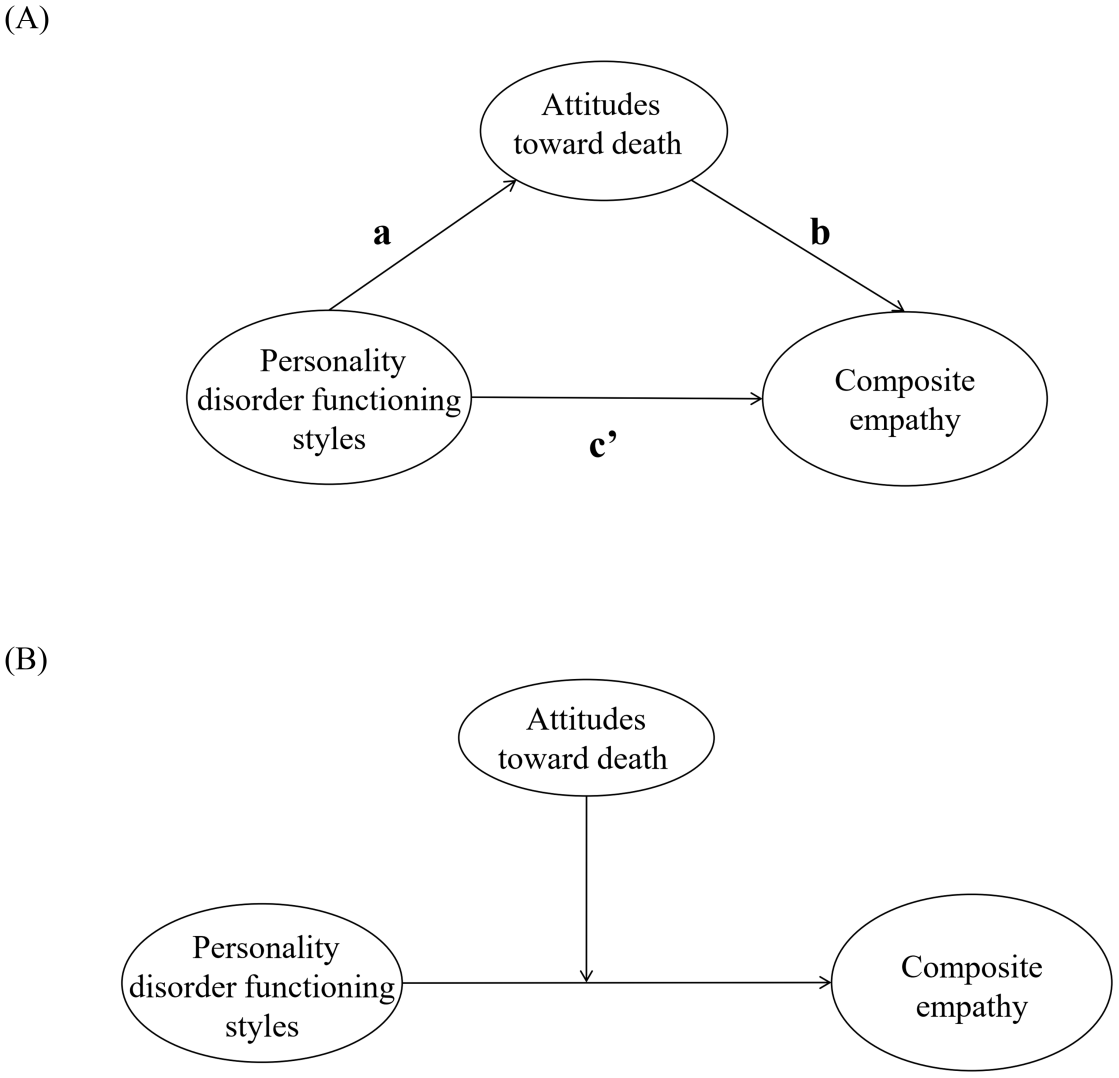
The theoretical **(A)** mediation and **(B)** moderation models in this study. Each model consists of 55 sub-models based on the potential mediating and moderating roles of five attitudes toward death between the 11 personality disorder styles and composite empathy in trainee nurses, respectively.

For hypothesis 1, Pearson correlation analysis was used to test the relationships between scores on the PERM, DAP-R, and JSE-HP total score in the trainee nurses. For hypotheses 2 & 3, least squares regression (bootstrap method) was used to analyze the mediating and moderating roles of death attitudes in PROCESS plug-in of SPSS 26.0 (51). Breusch-Pagan test was conducted to detect the heteroscedasticity between personality styles, attitudes toward death, and empathy, and VIF and tolerance were calculated for test of linearity. Then each of the 11 personality disorder functioning styles (i.e., Paranoid, Schizoid, Schizotypal, Antisocial, Borderline, Histrionic, Narcissistic, Avoidant, Dependent, Obsessive-compulsive, and Passive-Aggressive) was used as an independent variable, and the composite empathy was used as the dependent variable, as illustrated in **Figure 1**. To eliminate the multicollinearity effects between the variables, the data were centralized prior to analysis. 95% confidence intervals (CIs) were estimated by the bias-corrected bootstrapping procedure, and the number of iterations was set to 5000. A non-zero 95% CI for the interaction term and indirect effect indicated that specific attitudes toward death had a significant moderating and mediating role in the interaction terms and indirect effects.

## 3 Results

As showed in **Table 1**, 90.6% of the participants were female, 88.4% were between 21 and 23 years old, 88.3% were undergraduate students (followed by undergraduate and masters students). The demographic distribution was basically consistent with previous studies on Chinese trainee nurses (52, 53). In addition, no significant differences were observed in JSE-HP total scores between trainee nurses of different genders (Cohen’s d = 0.02) (also see **Supplementary Table 1** and results below) or education levels (η² = 0.003) (Ps > 0.05). However, higher scores were noted in younger nurses (r = -0.14, P = 0.001).

### 3.1 Test of correlations

**Table 2** showed Pearson’s correlations with age as a covariate. All PERM personality disorder functioning styles were negatively correlated with JSE-HP total scores of the trainee nurses (all Ps < 0.001). The study found positive correlations between all personality disorder functioning styles and DAP-R Fear of Death, Death Avoidance, Escape Acceptance, and Approach Acceptance (all Ps < 0.001), and negative correlations between all personality styles except the Obsessive-compulsive and Neutral Acceptance (all Ps < 0.001 except for schizoid personality disorder; P < 0.01 for schizoid personality disorder). Furthermore, Fear of Death, Death Avoidance, Approach Acceptance, and Escape Acceptance exhibited negative correlations, while Neutral Acceptance exhibited a positive correlation with the JSE-HP total scores of the trainee nurses (all Ps < 0.001). See **Supplementary Table 2** for more detailed descriptive statistics on scores of PERM, DAP-R, and JSE-HP among the participants. No other significant relationships were found between personality disorder functioning styles, death attitudes, and empathy in the trainee nurses.

**Table 2:** Correlations between the scores on the factors of Parker Personality Measure, the dimensions of Death Attitude Profile Revised, and the Jefferson Scale of Empathy-Health Professionals (JSE-HP) in trainee nurses (N = 614).

	Paranoid	Schizoid	Schizotypal	Antisocial	Borderline	Histrionic	Narcissistic	Avoidant	Dependent	Obsessive- compulsive	Passive- Aggressive	JSE-HP total score
Fear of Death	0.35***	0.23***	0.27***	0.30***	0.37***	0.32***	0.31***	0.40***	0.39***	0.18***	0.34***	-0.26***
Death Avoidance	0.18***	0.12***	0.11***	0.14***	0.20***	0.13***	0.13***	0.20***	0.23***	0.13***	0.17***	-0.13***
Escape Acceptance	0.43***	0.36***	0.40***	0.41***	0.49***	0.42***	0.39***	0.46***	0.43***	0.29***	0.44***	-0.40***
Approach Acceptance	0.38***	0.32***	0.32***	0.36***	0.41***	0.37***	0.35***	0.40***	0.39***	0.29***	0.36***	-0.26***
Neutral Acceptance	-0.25***	-0.10**	-0.26***	-0.27***	-0.23***	-0.22***	-0.24***	-0.16***	-0.23***	-0.02	-0.26***	0.41***
JSE-HP total score	-0.40***	-0.26***	-0.38***	-0.43***	-0.41***	-0.38***	-0.36***	-0.36***	-0.40***	-0.12***	-0.43***	–

*P < 0.05; **P < 0.01; ***P < 0.001.

### 3.2 Tests of mediating effects

There was no significant heteroscedasticity between personality styles and attitudes toward death or empathy (Ps > 0.05), while that between personality styles and empathy was significant (Ps < 0.01). Meanwhile, there was acceptable linearity between personality styles and attitudes toward death (VIFs = 1.95 ~ 8.92, tolerance = 0.11 ~ 0.51), between attitudes toward death and empathy (VIFs = 1.10 ~ 2.28, tolerance = 0.44 ~ 0.91), and between personality styles and empathy (VIFs = 1.95 ~ 8.92, tolerance = 0.11 ~ 0.51).

**Figure 2:**
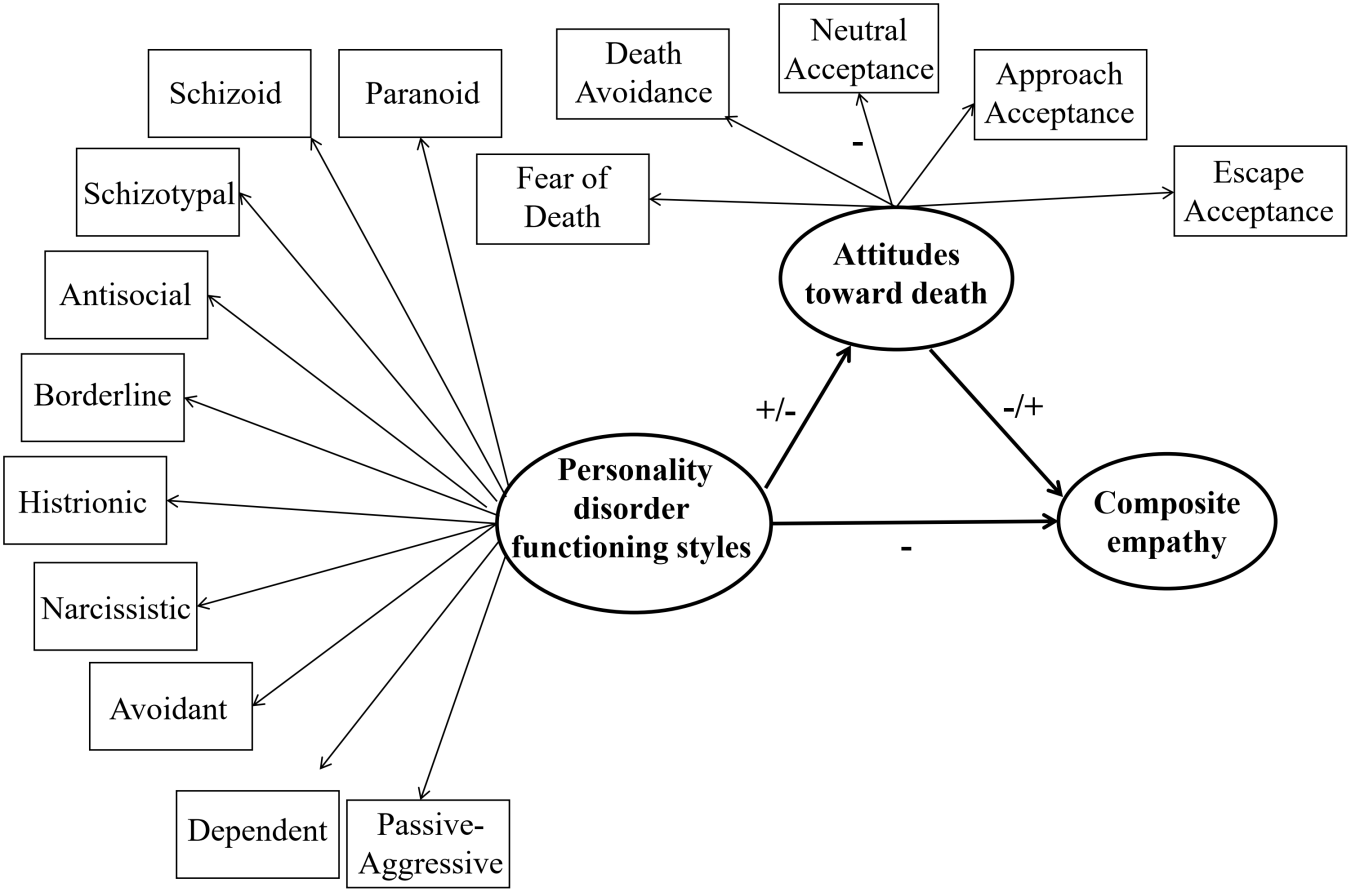
The schematic diagram of mediation model for trainee nurses.

**Table 3** displayed the results of the mediating effect with age as a covariate. When Fear of Death, Escape Acceptance, or Approach Acceptance was taken as the mediator, direct associations were found between all the personality disorder functioning styles (except for the Obsessive-compulsive) and JSE-HP total scores in trainee nurses (Ps < 0.05). The paths from Schizoid, Schizotypal, Histrionic, and Narcissistic styles to all five DAP-R death attitudes, as well as from these death attitudes to the JSE-HP total score, were included. Additionally, the paths from Paranoid, Antisocial, Borderline, Avoidant, Dependent, and Passive-Aggressive styles to all death attitudes except for Death Avoidance, as well as from these death attitudes to the JSE-HP total score, were also included. Significant associations were found between Obsessive-compulsive style and all death attitudes except for Neutral Acceptance, as well as between these death attitudes and JSE-HP total score (all Ps < 0.05). The bootstrap method revealed that the 95% confidence intervals of indirect effects in all models did not contain zero. This indicated that Fear of Death, Death Avoidance, Escape Acceptance, Approach Acceptance and Neutral Acceptance (–) partially mediated the negative associations of Schizoid, Schizotypal, Histrionic, and Narcissistic styles with empathy levels in the trainee nurses respectively. Fear of Death, Escape Acceptance, Approach Acceptance and Neutral Acceptance (–) partially mediate the negative associations of Paranoid, Antisocial, Borderline, Avoidant, Dependent, and Passive-Aggressive styles with their empathy levels respectively. Death Avoidance (-) partially mediated the negative association of Obsessive-compulsive style with their empathy levels. Moreover, Fear of Death, Escape Acceptance, and Approach Acceptance fully mediated the negative association of Obsessive-compulsive style with their empathy levels respectively (also see **Figure 2**).

**Table 3:** Mediating effects of death attitudes on the relationships between personality disorder functioning styles and the JSE-HP total scores in trainee nurses (N = 614).

Mediating Variable	Regression coefficients			Indirect effect	95%CI	c	a*b/c
	a	b	c’				
Paranoid style-death attitudes-JSE-HP total score							
Fear of Death	0.25***	-0.34***	-0.67***	-0.08	-0.14, -0.04	-0.73 ***	0.11
Neutral Acceptance	-0.10***	1.52***	-0.58***	-0.15	-0.22, -0.09	-0.73 ***	0.21
Approach Acceptance	0.36***	-0.24**	-0.65***	-0.09	-0.15, -0.03	-0.73 ***	0.12
Escape Acceptance	0.26***	-0.83***	-0.52***	-0.22	-0.29, -0.15	-0.73 ***	0.30
Schizoid style-death attitudes-JSE-HP total score							
Fear of Death	0.26***	-0.53***	-0.63***	-0.13	-0.21, -0.07	-0.75***	0.17
Death Avoidance	0.10**	-0.34**	-0.71***	-0.03	-0.08,>-0.01	-0.75***	0.04
Neutral Acceptance	-0.06*	1.79***	-0.64***	-0.11	-0.21, -0.02	-0.75***	0.15
Approach Acceptance	0.47***	-0.38***	-0.57***	-0.18	-0.26, -0.10	-0.75***	0.24
Escape Acceptance	0.33***	-1.05***	-0.40***	-0.35	-0.46, -0.25	-0.75***	0.47
Schizotypal style-death attitudes-JSE-HP total score							
Fear of Death	0.36 ***	-0.42***	-1.13***	-0.15	-0.24, -0.08	-1.28***	0.13
Death Avoidance	0.11**	-0.32*	-1.24***	-0.03	-0.08, >-0.01	-1.28***	0.03
Neutral Acceptance	-0.19***	1.54***	-0.99***	-0.30	-0.42, -0.18	-1.28***	0.25
Approach Acceptance	0.57***	-0.30***	-1.11***	-0.17	-0.27, -0.08	-1.28***	0.14
Escape Acceptance	0.45***	-0.88***	-0.88***	-0.40	-0.53, -0.28	-1.28***	0.33
Antisocial style-death attitudes-JSE-HP total score							
Fear of Death	0.22***	-0.35***	-0.73***	-0.08	-0.13, -0.03	-0.81***	0.10
Neutral Acceptance	-0.11***	1.46 ***	-0.65***	-0.16	-0.23, -0.10	-0.81***	0.20
Approach Acceptance	0.35***	-0.23**	-0.73***	-0.08	-0.14, -0.02	-0.81***	0.10
Escape Acceptance	0.25***	-0.80***	-0.61***	-0.20	-0.28, -0.14	-0.81***	0.24
Borderline style-death attitudes-JSE-HP total score							
Fear of Death	0.27***	-0.30 **	-0.70***	-0.08	-0.14, -0.03	-0.78***	0.11
Neutral Acceptance	-0.10***	1.53***	-0.63***	-0.15	-0.22, -0.09	-0.78***	0.21
Approach Acceptance	0.40***	-0.21**	-0.70***	-0.08	-0.15, -0.02	-0.78***	0.11
Escape Acceptance	0.30***	-0.77***	-0.54***	-0.23	-0.32, -0.15	-0.78***	0.33
Histrionic style-death attitudes-JSE-HP total score							
Fear of Death	0.38***	-0.38***	-1.02***	-0.15	-0.24, -0.07	-1.17 ***	0.13
Death Avoidance	0.12***	-0.28*	-1.17***	-0.03	-0.08, >-0.01	-1.17 ***	0.03
Neutral Acceptance	-0.15***	1.58***	-0.94***	-0.23	-0.36, -0.13	-1.17 ***	0.20
Approach Acceptance	0.59***	-0.27***	-1.01***	-0.16	-0.26, -0.06	-1.17 ***	0.14
Escape Acceptance	0.43***	-0.87***	-0.80***	-0.37	-0.50, -0.25	-1.17 ***	0.32
Narcissistic style-death attitudes-JSE-HP total score							
Fear of Death	0.26***	-0.41***	-0.69***	-0.11	-0.17, -0.05	-0.80***	0.14
Death Avoidance	0.08***	-0.30*	-0.77***	-0.02	-0.05, >-0.01	-0.80***	0.03
Neutral Acceptance	-0.12***	1.57***	-0.61***	-0.18	-0.27, -0.11	-0.80***	0.23
Approach Acceptance	0.40***	-0.29***	-0.69***	-0.12	-0.19, -0.05	-0.80***	0.15
Escape Acceptance	0.28***	-0.91***	-0.54***	-0.26	-0.35, -0.18	-0.80***	0.33
Avoidant style-death attitudes-JSE-HP total score							
Fear of Death	0.27***	-0.34**	-0.54***	-0.09	-0.16, -0.03	-0.63***	0.12
Neutral Acceptance	-0.06***	1.66***	-0.53***	-0.10	-0.16, -0.05	-0.63***	0.17
Approach Acceptance	0.36***	-0.27***	-0.53***	-0.10	-0.16, -0.04	-0.63***	0.15
Escape Acceptance	0.27***	-0.89***	-0.40***	-0.24	-0.32, -0.16	-0.63***	0.37
Dependent style-death attitudes-JSE-HP total score							
Fear of Death	0.29***	-0.30**	-0.66***	-0.09	-0.15, -0.03	-0.74***	0.12
Neutral Acceptance	-0.10***	1.55***	-0.60***	-0.15	-0.22, -0.09	-0.74***	0.20
Approach Acceptance	0.38***	-0.24**	-0.65***	-0.09	-0.16, -0.03	-0.74***	0.12
Escape Acceptance	0.27***	-0.84***	-0.52***	-0.23	-0.31, -0.15	-0.74***	0.31
Obsessive-compulsive style-death attitudes-JSE-HP total score							
Fear of Death	0.22***	-0.62***	-0.23	-0.14	-0.22, -0.07	-0.36**	/
Death Avoidance	0.12**	-0.40**	-0.32*	-0.05	-0.10,>-0.01	-0.36**	0.14
Approach Acceptance	0.47***	-0.47***	-.014	-0.22	-0.32, -0.13	-0.36**	/
Escape Acceptance	0.29***	-1.20***	‘-0.02	-0.35	-0.47, -0.24	-0.36**	/
Passive-Aggressive style-death attitudes-JSE-HP total score							
Fear of Death	0.27***	-0.32**	-0.79***	-0.09	-0.14, -0.03	-0.88***	0.10
Neutral Acceptance	-0.12***	1.48***	-0.71***	-0.17	-0.25, -0.10	-0.88***	0.19
Approach Acceptance	0.39***	-0.23**	-0.80***	-0.09	-0.15, -0.03	-0.88***	0.10
Escape Acceptance	0.30***	-0.78***	-0.65***	-0.23	-.032, -0.15	-0.88***	0.26

*P < 0.05, **P < 0.01, ***P < 0.001. Data without asterisks in both columns a and b were screened out since the absence of a mediating effect.

No other significant mediating effect of death attitude was found on the relationships between personality disorder functioning styles and empathy levels in the trainee nurses.

### 3.3 Tests of moderating effects

**Figure 3:**
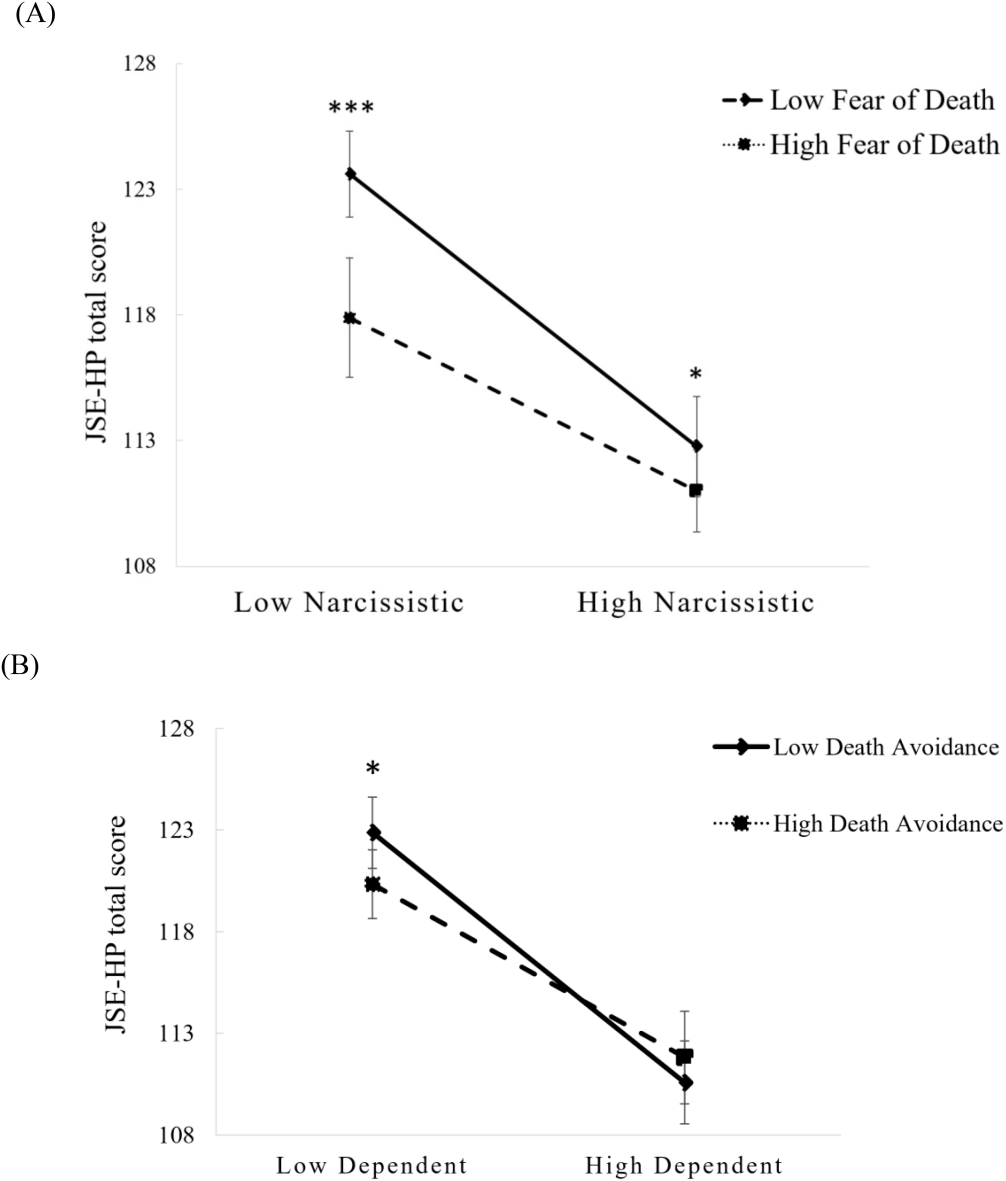
Interaction effects between **(A)** Fear of Death and Narcissistic style and **(B)** Death Avoidance and Dependent style on the JSE-HP total score of trainee nurses (N = 614). Error bands represent 95% confidence intervals. *P < 0.05; ***P < 0.001.

**Table 4** displayed the results of the moderating effect with age as a covariate. The JSE-HP total score was negatively predicted by both Narcissistic and Dependent styles (Ps < 0.001). Additionally, the interactions between Narcissistic style and Fear of Death, as well as between Dependent style and Death Avoidance, positively predicted the JSE-HP total score (Ps < 0.05). These results indicated that Fear of Death and Death Avoidance strengthened the negative predictions of Narcissistic and Dependent styles on empathy levels in trainee nurses respectively, as presented in **Figure 3**. No other significant moderating effect of death attitude was found upon the predictions of personality disorder functioning styles on their empathy levels.

**Table 4:** Moderating roles of death attitudes in the predictions of personality disorder functioning styles on the JSE-HP total scores in trainee nurses (N = 614).

Adjusting variables	β	S.E.	95%CI	R^2^
Model 1 Narcissistic style × death attitudes - JSE-HP total score				
				0.18***
Narcissistic	-0.71***	0.09	-0.88, -0.54	
Fear of Death	-0.38***	0.10	-0.57, -0.17	
Narcissistic × Fear of Death	0.03*	0.02	<0.01, 0.06	
Model 2 Dependent style × death attitudes - JSE-HP total score				
				0.18***
Dependent	-0.72***	0.07	-0.86, -0.58	
Death Avoidance	-0.08	0.14	-0.34, 0.19	
Dependent ×Death Avoidance	0.04*	0.02	<0.01, 0.07	

*P < 0.05, ***P < 0.001. Results of personality disorder functioning styles that did not significantly predict the empathy level in trainee nurses were not presented.

## 4 Discussion

In this study, we explored the relationships between personality disorder functioning styles, death attitudes, and empathy levels among trainee nurses. Regarding the influence of demographic information on composite empathy, we found no significant difference between genders, in contrast to some previous reports of lower levels in males (54, 55). We suggest that this may be due to the fact that the recently completed nursing education (including the nursing psychology curriculum) has improved the empathy of male trainee nurses (4) to a level similar to that of females. In general, we found that various personality disorder functioning styles were negatively associated with their empathy levels. Moreover, death attitudes like Fear of Death, Escape Acceptance, and Approach Acceptance mediated the relationships between all personality styles and their empathy levels; Neutral Acceptance mediated the relationships between all personality styles except Obsessive-compulsive and their empathy levels; besides, Death Avoidance mediated the relationship between some personality styles and their empathy levels. In addition, Fear of Death and Death Avoidance moderated the relationships between specific personality styles and their empathy levels. These results fully supported all of our hypotheses.

### 4.1 Correlations between the personality disorder functioning styles, death attitudes, and empathy levels in the trainee nurses

The current study revealed a negative association between each personality disorder functioning style and the empathy level of trainee nurses. Previous studies have shown that high level of non-adaptive personality traits, such as neuroticism, can be detrimental to the career development of nurses (56). Additionally, patients with personality disorders, such as schizoid, antisocial, narcissistic, or avoidant types, have been reported to have decreased empathetic concerns (30, 57–59). Such evidence supported our finding above. However, it is important to note that not all individuals with personality disorder lack empathy all the time. According to Ritter et al. (31), patients with narcissistic personality disorder underestimated their cognitive empathy and empathic behaviors on tests of empathic ability due to insufficient motivation, while in fact their cognitive empathy was normal, and it was their emotional empathy that was deficient. In addition, according to a review, patients with borderline personality disorder exhibited enhanced empathy in some social interaction situations, although these findings were contradicted by some other studies (60). Such evidence, on the one hand, conflicted with our results, and on the other hand, was suggesting that there were other factors influencing the relationship between personality disorder functioning styles and empathy, as we discussed below.

We found that attitudes toward death also influenced the empathy levels in trainee nurses. To be more specific, their JSE-HP total scores were negatively correlated with DAP-R Fear of Death, Death Avoidance, Approach Acceptance and Escape Acceptance, while positively correlated with Neutral Acceptance, in line with our previous findings on trainee nurses (61). Among the death attitudes, Fear of Death and Death Avoidance reflect negative thoughts and feelings about death, and a defense mechanism to keep death out of consciousness, respectively. On the contrary, Approach Acceptance and Escape Acceptance reflect a perception of death as a pathway to a blissful afterlife, and an escape from a painful existence, respectively. And unlike the attitudes above, Neutral Acceptance reflects a perception of death as a reality that is neither frightening nor desirable (32). Therefore, such findings implied that rational attitude like Neutral Acceptance of death instead of irrational ones, either fearing or desiring, was favorable for maintaining empathy at the right level. Although evidence showed that patients with schizophrenia with suicidal ideation had higher empathy levels compared to those without (62), indicating a positive correlation between an increase in tendency to death and higher empathy in patients with serious mental illness, other evidence supported the negative association between fear or avoidance of death and empathy in medical staff. For example, a study proved that the motivation of oncology nurses to care for terminally ill patients was negatively associated with death avoidance (63). Furthermore, we findings revealed that unlike empathy, the various personality disorder functioning styles were positively associated with the four irrational attitudes toward death mentioned above while negatively Neutral Acceptance, partly supported by the previous reports of increased fear of death in patients with narcissistic personality disorder, and higher suicidal ideation, which suggested higher desire for death, in patients with histrionic and antisocial personality disorders (40).

### 4.2 The mediating effect of death attitudes on the relationships between personality disorder functioning styles and empathy levels in the trainee nurses

Our findings that DAP-R Fear of Death, Death Avoidance, Escape Acceptance, Approach Acceptance, and Neutral Acceptance (–) partially mediated the negative associations of Schizoid, Schizotypal, Histrionic, and Narcissistic styles with the empathy levels of trainee nurses; and Fear of Death, Escape Acceptance, Approach Acceptance and Neutral Acceptance (–) partially mediated the negative associations of Paranoid, Antisocial, Borderline, Avoidant, Dependent, and Passive-Aggressive styles with their empathy levels indicated that the decrease in empathy among nurses with specific disordered personality traits could be attributed to their loss of neutrality towards death and the adoption of irrational attitudes towards it. Nurses were indeed a population that needed to be frequently exposed to high life risk and death. For example, studies have reported mortality rates as high as 40% in emergency and ICU units (64, 65). And such frequent exposure has been found to increase compassion fatigue in nurses (66), not to mention susceptible nurses who already have non-adaptive personality traits. Furthermore, classic study has shown that patients with schizophrenia spectrum disorders experienced social and emotional withdrawal when negative symptoms intensified and suicidal ideation emerged (67). It could be plausible that desire for death may reduce empathy in individuals with cluster A personality styles which associated with the schizophrenia continuum. This evidence supported the present findings. The risk of suicide and self-harm was also common in individuals with Cluster B and Cluster C personality disorders (68, 69), which might leave them with no room to empathize with others. For example, in narcissistic patients, when their narcissism was severely threatened, patients would develop increased approach or escape acceptance of death to get rid of shame, which in turn led them to suicidal thoughts and behaviors (70), and they have been reported to lack the capacity for real empathy (71). Similarly, borderline personality disorder with high suicide rates also had difficulty understanding the emotions of others, leading to an inability to meet the needs of others (72). However, a correlation has also been found between their empathic impairment and the severity of mood and psychotic symptoms (73), suggesting the mediation of factors other than attitudes toward death.

Furthermore, Death Avoidance partially mediated the negative association between Obsessive-Compulsive style and empathy, whereas Fear of Death, Approach Acceptance and Escape Acceptance fully mediated the negative association of Obsessive-Compulsive style and empathy, implying that Obsessive-Compulsive style could impair the empathy of trainee nurses partly through psychological defenses against death-related information, or entirely by causing them to be afraid of death, desire death, or use death as an escape from the suffering of reality. Individuals with obsessive-compulsive style often alleviate their internal anxiety by adhering to strict rules and striving for perfection (74), and their attention is narrowed (75). Death, on the other hand, symbolizes ultimate loss of control and uncertainty (76). When they are filled with the fear of death, it is easy for them to neglect the emotional needs of others because of their obsession with maintaining inner order. Conversely, when they can no longer cope with the pressures of reality, they may simply turn to death as the most effective way to solve problems (77), thus eliminating the need to focus on the suffering itself, which can also lead to a drop in empathy for others (78, 79).

### 4.3 The moderating effect of death attitudes on the relationships between personality disorder functioning styles and empathy levels in the trainee nurses

The findings further revealed that Fear of death and Death Avoidance also negatively moderated the JSE-HP total scores of nurses with Narcissistic or Dependent personality style, which suggested that such death attitudes further weakened the empathic competence of these nurses. Previous researches suggested that individuals diagnosed with narcissistic personality disorder exhibit lower acceptance of death (40) and a deficiency in empathy (80), they may engage in helping behaviors solely for the purpose of gaining attention rather than out of empathy or morality (71). On the other hand, patients with dependent personality disorder had high levels of insecurity and lack of self-confidence. When they perceived a relationship to be in jeopardy, they would resort to suicidal or abusive behavior to gain the attention of others (81). They were therefore prone to a lack of empathy.

### 4.4 Limitations and future directions

It is important to consider the limitations of the study. Firstly, it was a cross-sectional design, so the relationships between personality disorder functioning styles, attitudes toward death and empathy were inferred and need further validation through longitudinal studies. Secondly, although the sample of trainee nurses was obtained from a large general hospital, subsequent multicenter, cross-cultural validation of the findings is encouraged, particularly in light of recent evidence on the influence of culture on empathy (82–84). Thirdly, as the data were collected from self-report questionnaires, there may be a reporting bias. Fourthly, different aspects of empathy were not modelled separately as the study focused on the impact of different personality disorder styles and attitudes toward death on composite empathy. However, further modelling and validation of emotional, cognitive, and behavioral empathy respectively using other-assessment tools is warranted. Finally, we have primarily examined the impact of different personality disorder styles on empathy in early-career nurses, and further validation is also warranted in more experienced nurses and other healthcare professionals who have greater workloads and potentially greater exposure to death.

## 5 Conclusions

The study identified personality disorder functioning styles that could reduce empathy levels in trainee nurses by increasing fear and avoidance of or tendency to death, or by decreasing neutrality towards death; and that fear and avoidance of death exacerbated the decline in empathy in trainee nurses with narcissistic and dependent personality styles. Therefore, appropriate and timely education about life and death should be provided to nurses with specific personality dysfunctions to reduce their fear and avoidance of death, thereby increasing their composite empathy and improving the quality of health care.

## Data Availability

The raw data supporting the conclusions of this article will be made available by the authors, without undue reservation.

## Supplementary material

Supplementary Table 1 Demographic characteristics of the male and the two female subsamples.Using a random number table, two subsamples were randomly selected from the female trainee nurses. The student t-test showed that there was no significant difference between the male and the two female subsamples on the JSE-HP total score (using subsample 1: t = -0.25, P = 0.80, Cohen’s d = 0.05; using subsample 2: t = -0.79, P = 0.43, Cohen’s d = 0.14).

Supplementary Table 2 Descriptive statistics on scores of the Parker Personality Measure, the Death Attitude Profile Revised, and the Jefferson Scale of Empathy-Health Professionals (JSE-HP) among trainee nurses (N = 614).
