# Supplementary material for: Personality disorder functioning styles and empathy in trainee nurses: the mediating and moderating roles of death attitudes
Source: Front Psychiatry. 2025 Mar 27;16:1532940. doi: 10.3389/fpsyt.2025.1532940 (PMC12001526; doi:10.3389/fpsyt.2025.1532940)
Supplement: Supplementary Table 1 — Demographic characteristics of the male and the two female subsamples.Using a random number table, two subsamples were randomly selected from the female trainee nurses. The student t-test showed that there was no significant difference between the male and the two female subsamples on the JSE-HP total score (using subsample 1: t = -0.25, P = 0.80, Cohen’s d = 0.05; using subsample 2: t = -0.79, P = 0.43, Cohen’s d = 0.14). [file DataSheet1.docx]

**Supplementary Table 1** Demographic characteristics of the male and the two female subsamples.

| Variables | Male  N = 58 | Female subsample 1  n = 58 | Female subsample 2  n = 58 |
| --- | --- | --- | --- |
| Age (years) |  |  |  |
| 19 ~ 22 | 40 (69.0%) | 47 (81.0%) | 45 (77.6%) |
| 23 ~ 26 | 18 (31.0%) | 11 (19.0%) | 13 (22.4%) |
| Education level |  |  |  |
| College student and below | 2 (3.4%) | 3 (5.2%) | 7 (12.1%) |
| Undergraduate student | 56 (96.6%) | 55 (94.8%) | 51 (87.9%) |

| Variables | | Mean | S.D. | Minimum | Maximum | Skewness | Kurtosis |
| --- | --- | --- | --- | --- | --- | --- | --- |
| *Parker Personality Measure* | | | | | | | |
|  | Paranoid | 19.48 | 7.24 | 10.00 | 42.00 | 0.33 | -1.00 |
|  | Schizoid | 18.61 | 4.61 | 8.00 | 31.00 | -0.08 | -0.53 |
|  | Schizotypal | 9.09 | 3.93 | 5.00 | 20.00 | 0.58 | -0.99 |
|  | Antisocial | 18.28 | 7.11 | 10.00 | 37.00 | 0.54 | -0.95 |
|  | Borderline | 19.86 | 7.10 | 10.00 | 40.00 | 0.32 | -1.03 |
|  | Histrionic | 11.54 | 4.31 | 6.00 | 22.00 | 0.30 | -1.15 |
|  | Narcissistic | 15.01 | 6.05 | 8.00 | 30.00 | 0.41 | -1.16 |
|  | Avoidant | 22.05 | 7.45 | 10.00 | 41.00 | -0.03 | -1.01 |
|  | Dependent | 21.23 | 7.01 | 10.00 | 38.00 | 0.01 | -1.10 |
|  | Obsessive-compulsive | 15.14 | 4.23 | 6.00 | 27.00 | -0.34 | -0.41 |
|  | Passive-Aggressive | 18.00 | 6.47 | 9.00 | 37.00 | 0.25 | -1.11 |
| *Death Attitude Profile Revised* | | | | | | | |
|  | Fear of Death | 18.13 | 5.10 | 7.00 | 35.00 | -0.06 | -0.22 |
|  | Death Avoidance | 13.68 | 3.72 | 5.00 | 25.00 | -0.10 | 0.16 |
|  | Escape Acceptance | 12.03 | 4.33 | 5.00 | 25.00 | 0.24 | -0.48 |
|  | Approach Acceptance | 26.21 | 6.80 | 10.00 | 50.00 | -0.17 | 0.18 |
|  | Neutral Acceptance | 20.41 | 2.85 | 5.00 | 25.00 | -0.64 | 1.88 |
| *JSE-HP total score* | | 116.63 | 13.12 | 75.00 | 140.00 | -0.53 | 0.12 |

**Supplementary Table 2** Descriptive statistics on scores of the Parker Personality Measure, the Death Attitude Profile Revised, and the Jefferson Scale of Empathy-Health Professionals (JSE-HP) among trainee nurses (N = 614).
